# Atopic dermatitis and risk of autoimmune diseases: a systematic review and meta-analysis

**DOI:** 10.1186/s13223-021-00597-4

**Published:** 2021-09-25

**Authors:** Zhiyu Lu, Ni Zeng, Yuxin Cheng, Yihe Chen, Yueyue Li, Qian Lu, Qingyue Xia, Dan Luo

**Affiliations:** Department of Dermatology, The First Affiliated Hospital of Nanjing Medical University, Nanjing, China

## Abstract

**Background:**

Atopic dermatitis is the most common chronic inflammatory skin disease and presents a major public health burden worldwide. Recent observational studies revealed the potential association between atopic dermatitis with autoimmune disorders. However, there is no meta-analysis of the prevalence or incidence of autoimmune diseases in atopic dermatitis. Therefore, considering the potential clinical implications of these associations, we aimed to assess the risk of autoimmune diseases in patients with atopic dermatitis using this method.

**Methods:**

PubMed, Embase, and Web of Science were searched from inception to October, 2020. Observational studies which provided estimate effects with 95% CI or raw data were included. The quality of selected studies was evaluated using the Newcastle–Ottawa Scale. Odds ratio and relative risks were pooled using a random effects model and expressed with 95% confidence intervals.

**Results:**

Fourteen observational studies were included in this systematic review and meta-analysis. The random-effects meta-analysis of case–control and cross-sectional studies showed a significant association of atopic dermatitis with mutiple autoimmune diseases, including alopecia areata, celiac disease, Crohn’s disease, rheumatoid arthritis, systematic lupus erythematosus, ulcerative colitis and vitiligo. Furthermore, pooling of the results of cohort studies showed that patients with atopic dermatitis were more likely to develop these autoimmune diseases.

**Conclusion:**

Our meta-analysis showed that patients with atopic dermatitis were at higher risk of multiple autoimmune diseases including alopecia areata, celiac disease, Crohn’s disease, rheumatoid arthritis, systematic lupus erythematosus, ulcerative colitis and vitiligo. It is important for early detection of the affected group so that timely management can be initiated. Dermatologists and allergists should be aware of the autoimmune diseases in patients with atopic dermatitis and develop interventions if necessary. Also, limited by the present research, we still require more large-scale studies to further establish the association between atopic dermatitis and autoimmune diseases.

## Introduction

Atopic dermatitis (AD), also known as atopic eczema, is the most common chronic inflammatory skin disease characterized by intense itching and recurrent eczematous lesions. Nowadays, AD has become a major public health issues because of its high prevalence, considerable patient-burden as well as increased healthcare utilization. Meanwhile, AD brought huge economic burden to patients. To date, AD has affected at least 230 million people worldwide and being the leading cause of the non-fatal disease burden within skin conditions [1–4]. Potential mechanisms included epidermal barrier dysfunction, immune dysregulation and alteration of microbiome, which were modulated by genetic and environmental factors [5]. It has been reported that AD may be associated with allergic comorbidities including asthma, allergic rhinitis, food allergy and non-allergic comorbidities including psychological disorders, cardiovascular disorders, metabolic syndrome and multiple autoimmune diseases [6, 7].

Autoimmune disease is defined as a cluster of more than ninety diseases sharing a common pathogenesis of self-reactive adaptive immune response. Recent large-scale population-based studies have reported the association between AD and several autoimmune diseases. Paller [6] et al. had reviewed the association between AD with celiac disease, Crohn’s disease, ulcerative colitis and alopecia areata. However, this review was not conducted systematically and autoimmune diseases such as systematic lupus erythematosus and vitiligo were not included. So far, there were no systematic review and meta-analysis exploring the link between AD and multiple autoimmune diseases. Therefore, we performed this systematic review and meta-analysis based on the available literature to investigate the association between AD and autoimmune diseases.

## Methods

The protocol of this systematic review and meta-analysis was previously registered in the PROSPERO (CRD42020208929). This study was conducted in accordance with the Preferred Reporting Items for Systematic Reviews and Meta-Analyses (PRISMA) guidelines. We searched the PubMed, Embase, and Web of Science databases to identify all relevant studies that reported the association between AD and autoimmune diseases.

## Search strategy

PubMed, Embase, and Web of Science databases were searched from inception to October 2020 using the following search terms: (atopic dermatitis OR atopic eczema OR atopy) AND (autoimmune diseases OR autoimmune disorders OR autoimmunity OR autoantibody OR alopecia areata OR ankylosing spondylitis OR autoimmune hepatitis OR autoimmune hepatitis OR autoimmune thyroid diseases OR celiac disease OR Crohn’s disease OR diabetes OR Grave’s disease OR primary immune thrombocytopenia OR multiple sclerosis OR pernicious anemia OR rheumatoid arthritis OR Sjögren’s syndrome OR systematic lupus erythematosus OR systematic sclerosis OR ulcerative colitis OR urticaria OR vitiligo).

## Study selection

Studies were included if they met the following criteria: (i) cross-sectional or case–control studies reporting the prevalence of autoimmune diseases in patients with AD compared with controls, or raw data from which we could derive crude ORs with 95% CI; (ii) cohort studies reporting the incidence of autoimmune diseases in patients with AD compared with controls, or raw data from which we could derive crude RRs with 95% CI; (iii) Study groups had a clear definition of AD or atopic eczema, rather than dermatitis or eczema; (iv) Assessments of AD and autoimmune diseases included ICD, diagnosis of physicians, medical records which recorded physician-diagnosed AD or autoimmune diseases and questionnaire which included question such as “ever had clinician-diagnosed AD or autoimmine diseases”; (v) Articles were included if they were published in English, conducted in humans and available in full-text. Studies were excluded if they were reviews, meta-analyses, letters, case reports, comments and guidelines.

## Data extraction and quality assessment

We extracted surname of the first author, publication year, country/region, study design, demographic characteristics (numbers, age and female sex proportion) in the case and control groups, assessment used for AD and autoimmune diseases, risk estimates with 95% CI and corresponding adjustments for confounders. The methodological quality of selected studies was evaluated using the Newcastle–Ottawa Scale (NOS), which comprised three main items including sample selection, comparability and exposure/outcome. [8] Studies could be awarded a maximum of one star for each numbered item within the selection and exposure/outcome categories and a maximum of two stars for comparability category, with a score of 1–3, 4–6, and 7–9 represented as of low, intermediate, and high quality respectively. Two authors independently conducted quality assessments of the included studies. Discrepancies were discussed and resolved by the authors.

## Statistical analysis

Separate Random-effects meta-analysis with the inverse variance method was conducted for the association between AD with alopecia areata, celiac disease, Crohn’s disease, rheumatoid arthritis, systematic lupus erythematosus, ulcerative colitis and vitiligo. Random-effects model was applied because of a wide range of settings in different populations. We preferentially extracted and pooled adjusted risk estimates with 95% CI from primary studies. If these data were not available, we then derived crude risk estimates with 95% CI using raw data. Pooled ORs with 95% CI were calculated for cross-sectional and case–control studies and pooled RRs with 95% CI was calculated for cohort studies. Heterogeneity of included studies was assessed with the Cochran Q statistic, with a p value less than 0.10 suggesting evidence of heterogeneity. Further, the magnitude of heterogeneity was measured using the *I*^*2*^ statistic, with values of 25–49%, 50–75% and greater than 75% representing low, moderate and high heterogeneity respectively. All analyses were performed in STATA version 15.0 (Stata Corp, College Station, TX, USA) and Review Manager version 5.3 (The Cochrane Collaboration, London, UK).

## Result

**Fig. 1:**
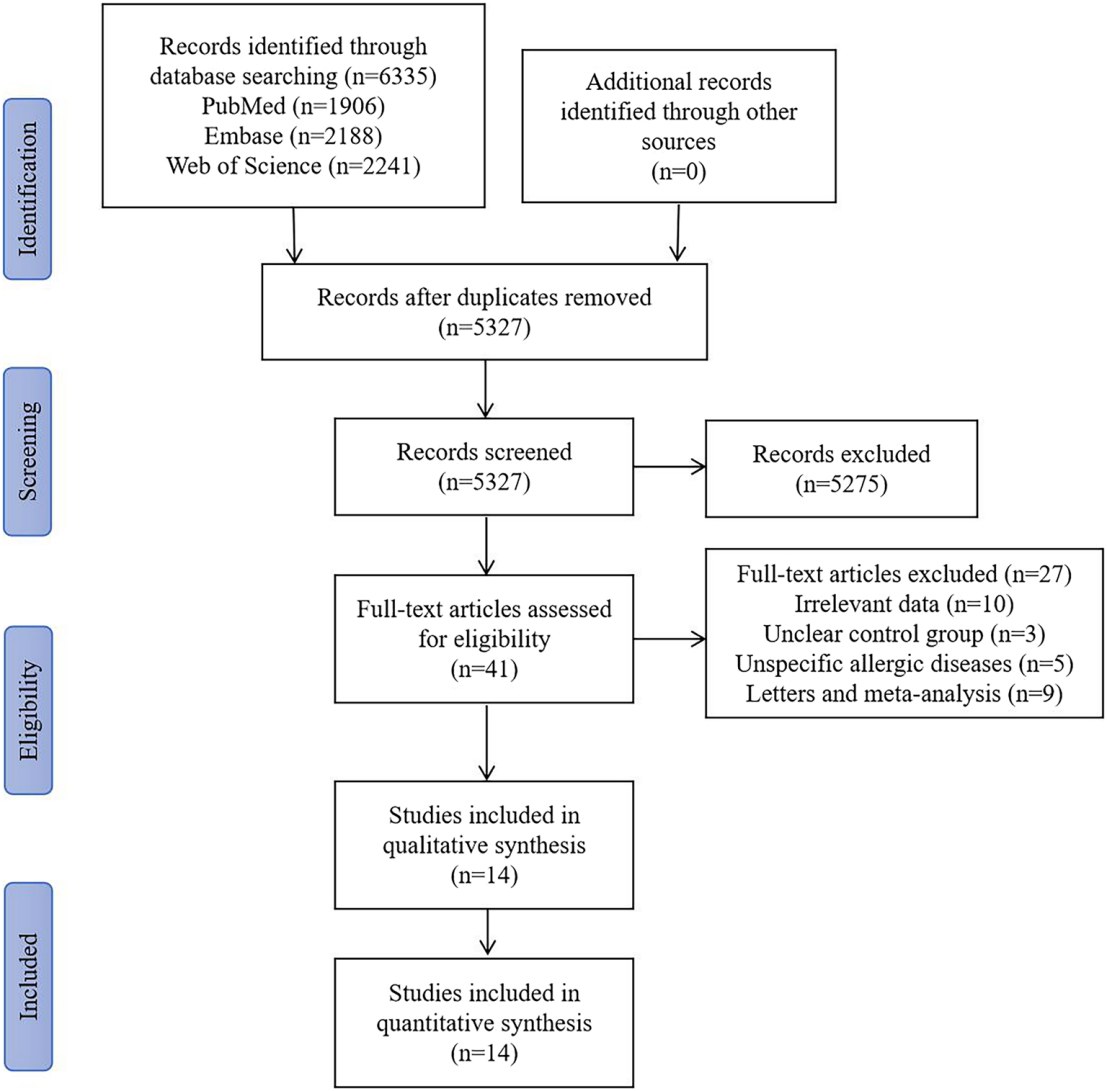
PRISMA flow chart of the search strategy

Figure 1 illustrated the process of study selection by the PRISMA flow chart. A total of 5327 articles were identified after removing the duplicates through the initial database search. After review of titles and abstracts, 5286 articles did not meet our inclusion criteria and 41 articles were left for full-text screening. After reading the full texts, 10 articles were excluded because of irrelevant data, three articles were excluded because of unclear control group, five articles were excluded because of unspecific allergic diseases, four articles were letters and five articles were meta-analysis. Eventually, 14 records were included in this qualitative and quantitative synthesis.

### Study characteristics

**Fig. 2:**
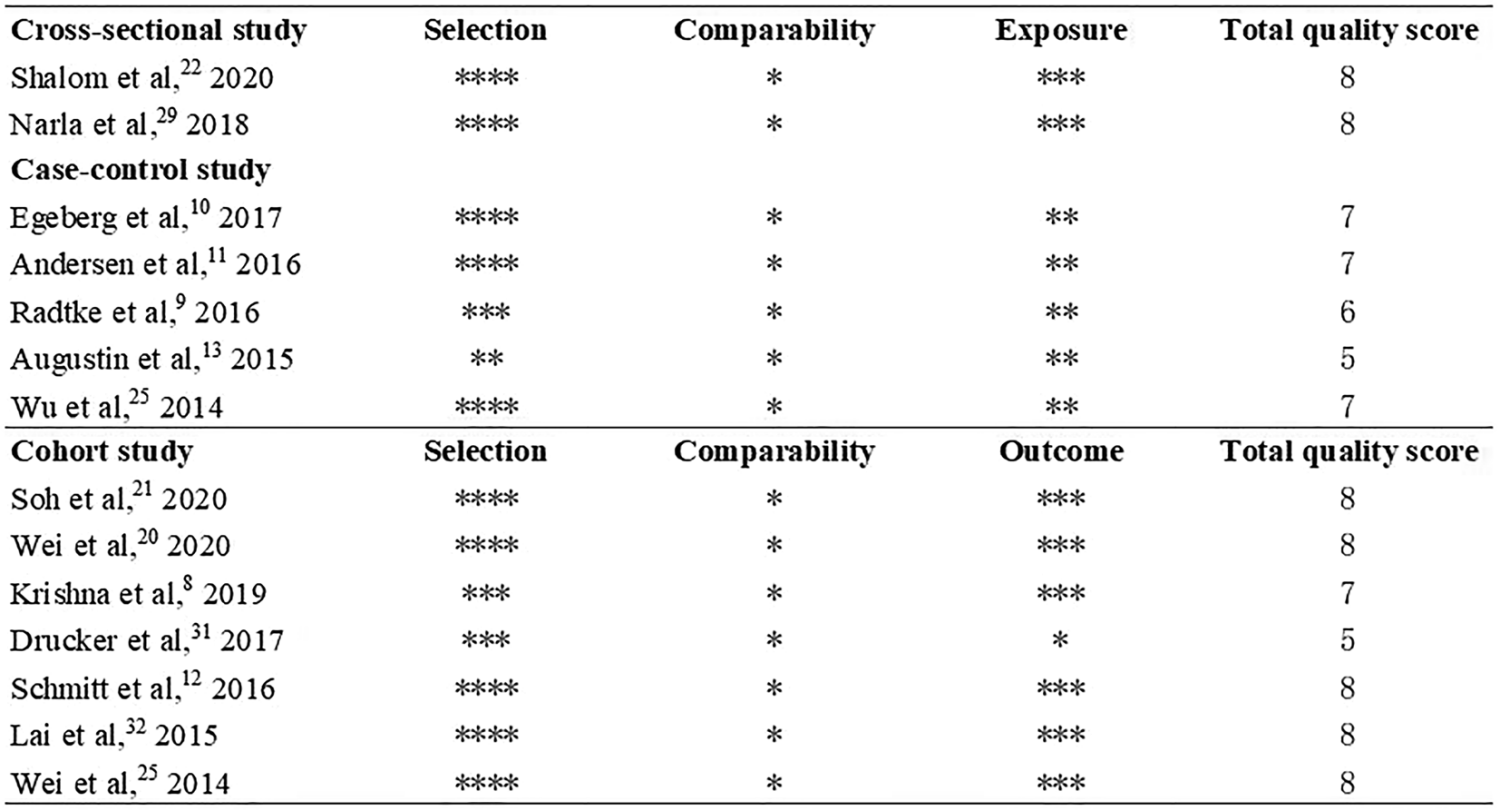
Methodological quality of selected studies by Newcastle–Ottawa Scale (NOS)

**Fig. 3:**
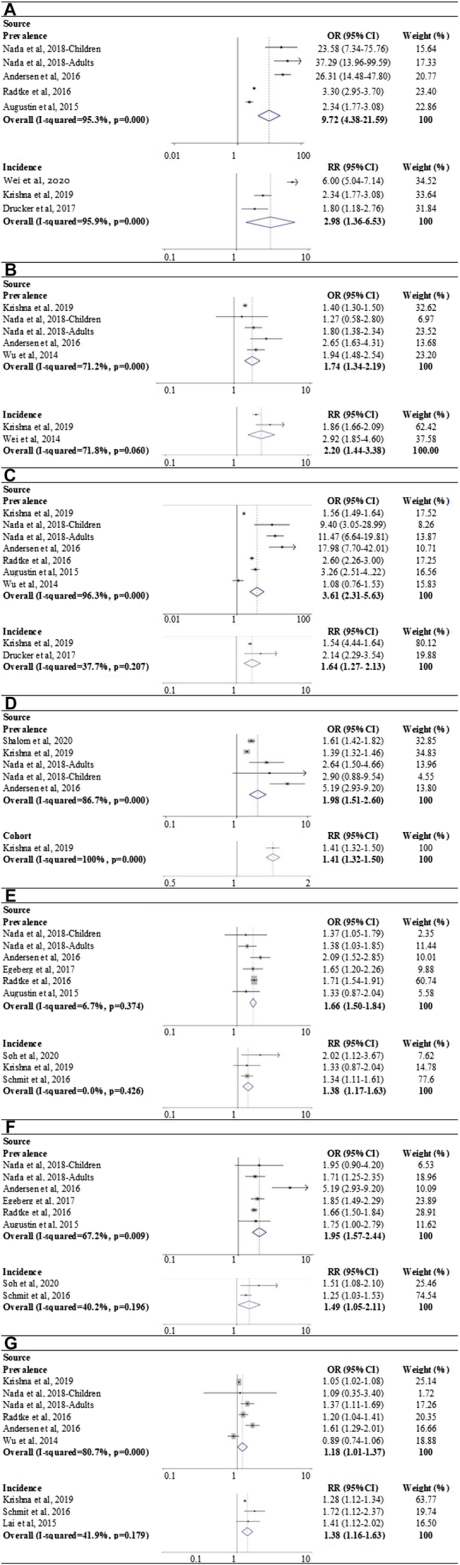
Forest plot for the prevalence and incidence of mutiple autoimmune diseases in AD compared to controls

Table 1 detailed the characteristics of included studies. A total of 14 studies were included in this review. The publication year ranged from 2014 to 2020. 90,568,121 patients with AD and 101,324,307 controls were included. For study location, six studies were from Europe [9–14], five studies were from Asia [15–19] and three studies [20–22] were from North America. In addition, the assessments of AD and autoimmune diseases included ICD-9 or 10, diagnosis of dermatologists or physicians, medical records and questionnaire by self-reported. Risk of bias assessment revealed using the Newcastle–Ottawa Scale revealed that 11 included studies were high quality and three studies were moderate quality (Fig. 2). Figure 3 provided the forest plot for the prevalence and incidence of mutiple autoimmune diseases in patients with AD compared to control patients. The effect size and corresponding adjustment was also provided (Table 2).

**Table 1:** Characteristics of included studies (part 1)

Study	Location	Study design	Assessment of AD	Assessment of AID	Cases (AD)			Control		
					Total, n	Age (Mean ± SD), y	Female (%)	Total, n	Age (Mean ± SD), y	Female (%)
Shalom et al. [17] 2020	Israel	Cross-sectional	Dermatologist	Gastroenterologist	116,816	21.4 ± 20.1	61,892 (53.0)	116,812	21.4 ± 20.1	61,889 (53)
Narla et al. [20] 2018	USA	Cross-sectional	ICD-9	ICD-9	87,053,155	NA	NA	87,033,669	NA	NA
Egeberg et al. [11] 2017	Denmark	Case–control	ICD-10	ICD-10	7,937	34.3 ± 14.1	4,901 (61.8)	79,370	34.3 ± 14.1	49,010 (61.8)
Andersen et al. [12] 2016	Denmark	Case–control	ICD-10	ICD-10	8,112	42.4 ± 15.2	4,982 (61.4)	40,560	42.4 ± 15.2	24,910 (61.4)
Radtke et al. [10] 2016	Germany	Case–control	ICD-10	ICD-10	48,140	NA	NA	1,301,531	NA	NA
Augustin et al. [14] 2015	Germany	Case–control	ICD-10	ICD-10	30,354	NA	NA	262,827	NA	NA
Wu et al. [18] 2014	China Taiwan	Case–control	ICD-9	ICD-9	41,950	34.7 ± 23.8	22,775 (54.3)	167,800	34.7 ± 23.8	91,100 (54.29)
Soh et al. [16] 2020	Korea	Cohort	ICD-10	ICD-10	1,557,435	NA	NA	8,366,086	NA	3,618,584 (43.2)
Wei et al. [15] 2020	China Taiwan	Cohort	ICD-9	ICD-9	40,307	20.6 (8.8–37.9)	22,546 (55.9)	161,228	20.6 (8.8–37.9)	90,184 (55.9)
Krishna et al. [9] 2019	UK	Cohort	Medical records	Medical records	1,393,570	29.8 (25.3)	754,293 (54.1)	2,170,618	32.7 (25.5)	1,167,859 (53.8)
Drucker et al. [21] 2017	USA	Cohort	Self-reported	Self-reported	9,234	54.4 (4.6)	9,234 (100)	78,172	54.5 (4.7)	78,172 (100)
Schmitt et al. [13] 2016	UK	Cohort	ICD-10	ICD-10	49,847	NA	29,231 (58.6)	605,968	NA	295,510 (48.8)
Lai et al. [22] 2015	Canada	Cohort	ICD-9	ICD-9	18,907	NA	NA	170,238	46.6 ± 17.4	92,736 (54.5)
Wei et al. [19] 2014	China Taiwan	Cohort	ICD-9	ICD-9	192,357	3.2 ± 0.04	89,749 (46.7)	769,428	3.3 ± 0.04	358,995 (46.7)

*ICD* international classification of diseases, *AD* atopic dermatitis, *AID* autoimmune diseases, *NA* not available

**Table 2:** Characteristics of included studies (part 2)

Study	Effect size (95% CI)	Adjusted for
Shalom et al. [17] 2020	Celiac disease: aOR = 1.609 (1.42–1.82)	Age, sex, smoking status and primary care visits
Narla et al. [20] 2018	32 specific autoimmune diseases: adult (aOR 1.45 1.32–1.58); children (aOR 2.08 1.73–2.5)	Age, sex, race/ethnicity, and insurance status
Egeberg et al. [11] 2017	CD: aOR = 1.65 (1.20–2.26); UC: aOR = 1.85 (1.49–2.29)	Age, sex, socioeconomic status, and number of dermatologist visits
Andersen et al. [12] 2016	22 specific autoimmune diseases: overall effect not available	Age, sex, smoking, socioeconomic status, and number of dermatologist visits
Radtke et al. [10] 2016	CD: OR = 1.71 (1.54–1.91); UC: OR = 1.66 (1.50–1.84); AA: OR = 3.30 (2.95–3.70); vitiligo: OR = 2.60 (2.26–3.00)	NA
Augustin et al. [14] 2015	CD: OR = 1.33 (0.87–2.04); UC: OR = 1.75 (1.10–2.79); AA: OR = 2.34 (1.77–3.08); vitiligo: OR = 3.26 (2.51–4.22)	NA
Wu et al. [18] 2014	T1DM: aOR = 1.00 (0.90–1.13); PA: aOR = 0.54 (0.20–1.41); RA: aOR = 0.89 (0.74–1.06); SLE: aOR = 1.94 (1.48–2.54); vitiligo: aOR = 1.08 (0.76–1.53); ATD: aOR = 0.93 (0.76–1.14)	Age, gender, number of healthcare visits, presence of allergic rhinitis, asthma and the autoimmune disorders listed in this table
Soh et al. [16] 2020	CD: aRR = 2.02 (1.12–3.67); UC: aRR = 1.51 (1.08–2.10)	Age, sex, residence, smoking history, alcohol consumption, regular exercise, income, body mass index, diabetes, hypertension, and dyslipidemia
Wei et al. [15] 2020	AA: aRR = 6.00 (5.04–7.14)	
Krishna et al. [9] 2019	CD: aRR = 1.33 (0.87–2.04); UC: aRR = 1.75 (1.10–2.79); AA: aRR = 2.34 (1.77–3.08); vitiligo: aRR = 1.67 (1.50–1.87); Sjogrens syndrome: aRR = 1.52 (1.22–1.88); RA: aRR = 1.31 (1.22–1.41); PA: aRR = 1.23 (1.11–1.35); MG: aRR = 1.04 (0.76–1.41); celiac disease: aRR = 1.37 (1.24–1.52); ATD: aRR = 1.14 (1.00–1.30); multiple sclerosis: aRR = 1.15 (0.96–1.37); SLE: aRR = 2.40 (1.98–2.91)	Age, sex, socio-economic status, smoking status, BMI and ethnicity
Drucker et al. [21] 2017	AA: aRR = 1.80 (1.18–2.76); vitiligo: aRR = 2.14 (2.29–3.54)	Age, BMI, alcohol intake, physical activity, cigarette smoking status and history of postmenopausal hormone replacement use
Schmitt et al. [13] 2016	CD: aRR = 1.34 (1.11–1.61); UC: aRR = 1.25 (1.03–1.53); RA: aRR = 1.72 (1.12–2.37); T1DM: aRR = 0.72 (0.53–0.998)	Age, sex, socioeconomic status, access to health care and health care utilization behavior
Lai et al. [22] 2015	RA: aRR = 1.41 (0.98–2.02)	Age, sex, urbanization level, income and diabetes mellitus
Wei et al. [19] 2014	SLE: aRR = 2.92 (1.85–4.60)	Sex, age, urbanization, and mutual five allergic diseases (asthma, allergic rhinitis, allergic conjunctivitis, atopic dermatitis and urticaria)

*AA* alopecia areate, *CD* Crohn’s disease, *RA* rheumatoid arthritis, *SLE* systematic lupus erythematosus, *UC* ulcerative colitis, *aOR* adjusted odds ratio, *CI* confidence interval, *aRR* adjusted relative risk

### Association between AD and alopecia areata

Four studies compared the prevalence of alopecia areata in AD patients and controls [10, 12, 14, 20]. Meta-analysis showed a significantly higher prevalence of alopecia areata in AD patients compared to the controls, with a pooled OR of 9.72 (95% CI 4.38–21.59). There was high statistical heterogeneity (*I*^*2*^ = 95.2%, p = 0.000). Further, the pooling of the results from three cohort studies that reported the incidence showed that AD increased the risk of developing alopecia areata (RR 2.98 95% CI 1.36–6.53) [9, 15, 21]. High statistical heterogeneity was also detected (*I*^*2*^ = 95.9%, p = 0.000) (Fig. 3A and Table 3).

**Table 3:** Results of pooling odds ratio and risk ratio of includied studies

Type of autoimmune disease	Cross-sectional/case–control studies			Cohort studies		
	Studies(n)	Case/control(n)	OR (95% CI)	Studies(n)	Case/control(n)	RR (95% CI)
Alopecia areata	4	87,139,761/88,638,587	9.72 (4.38–21.59)	3	1,443,111/2,410,018	2.98 (1.36–6.53)
Systematic lupus erythematosus	4	88,496,787/89,412,647	1.74 (1.34–2.19)	2	1,585,927/2,940,046	2.20 (1.44–3.38)
Vitiligo	6	88,529,196/ 89,676,032	3.61 (2.31–5.63)	2	1,402,804/2,248,790	1.64 (1.27–2.13)
Celiac disease	4	88,571,653/89,361,659	1.98 (1.51–2.60)	1	1,393,570/2,170,618	1.41 (1.32–1.50)
Crohn’s disease	5	87,101,613/87,416,984	1.66 (1.50–1.84)	3	3,000,852/2,776,586	1.38 (1.17–1.63)
Ulcerative colitis	5	87,101,613/87,416,984	1.95 (1.57–2.44)	2	1,607,282/8,972,054	1.49 (1.05–2.11)
Rheumatoid arthritis	5	88,527,141/89,675,474	1.18 (1.01–1.37)	3	1,462,324/2,946,824	1.38 (1.16–1.63)

### Association between AD and systematic lupus erythematosus

The prevalence of systematic lupus erythematosus in AD and controls was reported in four studies [9, 12, 18, 20]. Patients with AD had a higher prevalence of systematic lupus erythematosus compared to controls, with an OR of 1.74 (95% CI 1.34–2.19 *I*^*2*^ = 71.2%, p = 0.008). Similarly, the incidence of systematic lupus erythematosus in AD and controls was reported in two cohort studies, with an average RR of 2.20 (95% CI 1.44–3.38, *I*^*2*^ = 71.8%, p = 0.060), indicating an increased risk of developing systematic lupus erythematosus in AD patients (Fig. 3B and Table 3) [9, 19].

### Association between AD and vitiligo

Six studies revealed the increased prevalence of vitiligo in AD compared to controls (OR 4.52 95% CI 2.51–8.13, *I*^*2*^ = 96.3%, p = 0.000) [9, 10, 12, 14, 18, 20]. Two cohort studies reported the elevated incidence of vitiligo in AD compared to controls, which showed AD patients were more likely to develop vitiligo (RR 1.64 95% CI 1.27–2.13, *I*^*2*^ = 37.7%, p = 0.207) (Fig. 3C) [9, 21].

### Association between AD and celiac disease

Prevalence of celiac disease in AD and controls was reported in four studies [9, 12, 17, 20]. Meta-analysis showed a higher prevalence of celiac disease in AD compared to controls. The synthesized OR was 1.98 (95% CI 1.51–2.60) with high heterogeneity (*I*^*2*^ = 86.7%, p = 0.000). Only one cohort reported an increased incidence of celiac disease in AD, with a RR of 1.41 (95% CI 1.32–1.50) (Fig. 3D and Table 3) [9].

### Association between AD and Crohn’s disease

Pooling result of five studies estimated an elevated prevalence of Crohn’s disease in AD compared to controls, with an average OR of 1.66 (95% CI 1.50–1.84, *I*^*2*^ = 6.7%, p = 0.374) [10–12, 14, 20]. Three cohort studies further detected an increased incidence of Crohn’s disease in AD, with a pooled RR of 1.38 (95% CI 1.17–1.63, *I*^*2*^ = 0.0%, p = 0.426) (Fig. 3E and Table 3), indicating that patients with AD had higher risk of developing Crohn’s disease [9, 13, 16].

### Association between AD and ulcerative colitis

A total of five studies [10–12, 14, 20] and two cohort studies [13, 16] were included in this analysis. The prevalence and incidence were higher in AD compared with control, with a pooled OR of 1.95 (95% CI 1.57–2.44, *I*^*2*^ = 67.2%, p = 0.009) and a pooled RR of 1.49 (95% CI 1.05–2.11, *I*^*2*^ = 40.2%, p = 0.196) respectively (Fig. 3F and Table 3). These results showed that AD increased the risk of developing ulcerative colotis.

### Association between AD and rheumatoid arthritis

Five studies [9, 10, 12, 18, 20] reported the prevalence and three cohort studies [9, 13, 22] reported the incidence of rheumatoid arthritis in patients with AD. The prevalence of rheumatoid arthritis was higher in AD compared to control (OR 1.18 95% CI 1.01–1.37, *I*^*2*^ = 80.7%, p = 0.000). The incidence was also higher in AD compared to control, with an average RR of 1.38 (95% CI 1.16–1.63, *I*^*2*^ = 41.9%, p = 0.179) (Fig. 3G and Table 3), which meant AD could increased the risk of developing rheumatoid arthritis.

## Discussion

To date, the mechanism of association between AD and autoimmune diseases is not clear. Several possible explanations should be noted. Atopic dermatitis is characterized by cutaneous inflammation due to abnormalities of innate immune responses and autoimmune diseases result from a complex dysregulation of innate and adaptive immunity. Common immunologic pathways might be shared in both atopic dermatitis and autoimmune diseases. Interestingly, TH1/TH17 signaling have been presented in both atopic dermatitis and several autoimmune diseases, including SLE, IBD and RA [23, 24]. Genetic association might be another possible explanation. A recent meta-analysis of genome-wide association studies based on 21,000 cases and 95,000 controls identified 10 new susceptibility loci for atopic dermatitis. All novel susceptibility loci were associated with autoimmune regulation and there were substantial genetic overlaps with multiple autoimmune diseases [25]. Additionally, it has been reported that AD, vitiligo responded to inhibition of the JAK-STAT pathway [26, 27]. Despite these possible common pathophysiology, the correlation of AD with autoimmune diseases has been rarely studied. Identification of common mechanisms between AD and autoimmune diseases might provide common therapeutic approaches for both diseases.

This is the first and most comprehensive systematic review and meta-analysis to evaluate the prevalence and incidence between AD and multiple autoimmune diseases, which included alopecia areata, celiac disease, Crohn’s disease, rheumatoid arthritis, systematic lupus erythematosus, ulcerative colitis and vitiligo. A previous systematic review and meta-analysis conducted by Mohan et al. [28] found a higher risk of AD in patients with either vitiligo (OR 7.82, 95% CI 3.06–20.00) or alopecia areata (OR 2.57, 95% CI 2.25–2.94). However, it was not clear whether AD was at increased risk of vitiligo or alopecia areata. In our meta-analysis, we further explore the association between AD with vitiligo and alopecia areata. Acharya et al. [29] further explored the prevalence and incidence of vitiligo in AD. Results revealed significant association between AD and vitiligo (OR 3.21, 95% CI 1.90–5.43). In a cohort included in this meta-analysis, the prevalence and incidence of autoimmune diseases were both provided. However, the prevalence of the included cohort was not pooled in the meta-analysis. We then calculated the OR with 95% CI and added it to the pooled meta-analysis. In addition, the other two meta-analysis suggested a bidirectional relationship between AD and inflammatory bowel diseases including Crohn’s disease and ulcerative colitis [30, 31].

### Limitation

Several limitations should be emphasized. Firstly, limited by the number of included studies, subgroup analyses stratified by age, sex, subset and severity of AD were not performed. Secondly, we found minimal publication bias in two meta-analyses, which might result from flawed methodologic design in studies with small sample size. Thirdly, though the fully adjusted estimate effects with 95% CI were extracted, the adjusted confounding factors (such as smoking, use of medication and socioeconomic status) were inconsistent among the included studies. Several included studies were of small sample size with moderate methodological quality. The assessments of AD and autoimmune disorders in part of studies were based on questionnaires, which might lead to misdiagnosis of these diseases. Additionally, only English articles were included due to the authors’ language restriction and studies in other languages might be missed, which could lead to potential bias in our results.

### Future practice

Further research is needed to identify whether the subset and severity of AD are at elevated risk for autoimmune diseases. In addition, not all autoimmune diseases were analyzed, therefore we require more large-scale studies to further establish the association between AD and more autoimmune diseases. Future research is also needed to assess the risk factors such as medication treatments for comorbid autoimmune diseases in patients with AD. The underlying mechanism of the association between AD and autoimmune comorbidities should also be established. Moreover, clinicians especially allergists and dermatologists should be aware of the autoimmune diseases in patients with AD and develop interventions to screen and monitor for them.

## Conclusion

We performed a systematic review and meta-analysis of observational studies which included 90,568,121 patients with AD and 101,324,307 controls to examine the association between AD with multiple autoimmune diseases. It was founded that AD was associated with increased risks of alopecia areata, celiac disease, Crohn’s disease, rheumatoid arthritis, systematic lupus erythematosus, ulcerative colitis and vitiligo. However, our meta-analyses were based on the limited studies which were of cross-sectional, case–control and retrospective cohort designs. Therefore, more large-scale prospective clinical trials and mechanistic studies were warranted to further elucidate the association between AD and more autoimmune diseases.

## Data Availability

Not applicable.
